# Copper Homeostasis as a Therapeutic Target in Amyotrophic Lateral Sclerosis with *SOD1* Mutations

**DOI:** 10.3390/ijms17050636

**Published:** 2016-04-28

**Authors:** Eiichi Tokuda, Yoshiaki Furukawa

**Affiliations:** Laboratory for Mechanistic Chemistry of Biomolecules, Department of Chemistry, Keio University, Yokohama 223-8522, Japan; tokuda@chem.keio.ac.jp

**Keywords:** amyotrophic lateral sclerosis, Cu,Zn-superoxide dismutase, copper homeostasis

## Abstract

Amyotrophic lateral sclerosis (ALS) is a lethal neurodegenerative disease affecting both upper and lower motor neurons, and currently, there is no cure or effective treatment. Mutations in a gene encoding a ubiquitous antioxidant enzyme, Cu,Zn-superoxide dismutase (SOD1), have been first identified as a cause of familial forms of ALS. It is widely accepted that mutant SOD1 proteins cause the disease through a gain in toxicity but not through a loss of its physiological function. SOD1 is a major copper-binding protein and regulates copper homeostasis in the cell; therefore, a toxicity of mutant SOD1 could arise from the disruption of copper homeostasis. In this review, we will briefly review recent studies implying roles of copper homeostasis in the pathogenesis of *SOD1*-ALS and highlight the therapeutic interventions focusing on pharmacological as well as genetic regulations of copper homeostasis to modify the pathological process in *SOD1*-ALS.

## 1. Introduction

Amyotrophic lateral sclerosis (ALS) is the most common adult-onset form of neuromuscular disorder characterized by the loss of motor neurons in motor cortex, brainstem, and spinal cord. ALS is clinically heterogeneous in the age at disease onset, the initial site of symptoms, and the rate of disease progression [1]. Despite such clinical heterogeneity, patients generally undergo muscle weakness, atrophy, paralysis, and eventually premature death due to respiratory failure with a median survival period of three years after the onset of initial symptoms. Riluzole is the only therapeutic option available for ALS, but its therapeutic effect is limited: it can extend survival by a few months but without any improvement in muscle function [2]. While there is a need to develop new approaches for treatment of ALS, the predominant proportion of ALS cases (>90%) is sporadic with no genetic predisposition [3], which makes it difficult to identify a specific target for developing therapeutics. Instead, the pathological character of sporadic ALS (sALS) is partly overlapped with that of familial ALS constituting the remaining 5%–10% cases [1]. Among several genes responsible for familial ALS (fALS), mutations in a gene encoding Cu,Zn-superoxide dismutase (SOD1) have been the most intensely studied [4], and, therefore, experimental data accumulated on fALS with *SOD1* mutations (*SOD1*-ALS) would provide useful insight into the therapeutic development for the treatment of this devastating disease.

## 2. A Gain-of-Toxicity of Mutant SOD1 in a Pathomechanism of *SOD1*-ALS

To date, over 180 different mutations in *SOD1* gene have been identified as a cause of fALS [5]. and are scattered throughout the entire sequence of the protein. SOD1 is a metalloenzyme that binds copper and zinc ions for removing a reactive oxygen species, superoxide anion [6]. Mutation-induced reduction in the enzymatic activity as well as the metal binding affinity of SOD1 had been first proposed to be pathogenic [7,8] but was later found not to be required for ALS; complete deletion in *SOD1* gene in mice did not develop an ALS-like phenotype [9]. Furthermore, SOD1 proteins with different pathogenic mutations exhibited distinct affinity for metal binding *in vitro*, which is little correlated with disease onset/duration *in vivo* [10,11,12]. For example, mutations in two of the ligands for copper binding, H46R and H48Q, completely abolish the copper-binding affinity [13,14] and thus negate the enzymatic activity of SOD1, but fALS with H46R mutation showed significantly slower disease progression (6–30 years) [15] than that in fALS with H48Q mutation (~1 year) [16]. Also, G93A mutation almost fully retains the copper-binding ability of SOD1 *in vitro* [10] but showed severe phenotype (~2 years of disease duration) [17]. It is hence generally accepted that mutations in *SOD1* are involved in the disease through a gain of toxicities but not a loss of its physiological functions. Currently, many hypotheses for toxicities of mutant SOD1 proteins have been proposed including increased oxidative stress [18], mitochondrial dysfunction [19], glutamate excitotoxicity [20], accumulation of protein aggregates [21], perturbation of proteostasis [22,23], non-neuronal cell autonomous toxicity [24], and metal homeostasis [25]. Among those, the disruption of metal homeostasis has long been debated as a possible pathomechanism of *SOD1*-ALS, because SOD1 is a metalloprotein binding copper and zinc ions. In this review, therefore, we will focus on possible roles of copper dyshomeostasis in the pathomechanism of *SOD1*-ALS.

## 3. Intracellular Regulation of Copper Ions for SOD1 Activation

Copper ion is an essential trace element for various physiologies; the majority of copper ions is absorbed in the small intestine and delivered to the liver and kidneys. In the liver, copper ions are predominately bound to ceruloplasmin and then released into the blood circulation [26]. In serum, 65%–90% of copper ions is tightly bound to ceruloplasmin [26], which cannot, however, cross the blood–brain barrier (BBB); instead, copper ions are transported into the central nervous system (CNS) as a free form via cerebral capillaries comprising BBB [27]. Given that the cerebral capillaries are largely covered by the foot of astrocytes, astrocytes are considered as the first parenchyma cells in the CNS to encounter copper ions that cross the BBB [28]. Actually, astrocytes are known to specifically express membrane-anchored ceruloplasmin [29] and are also considered to have high capacity of copper ions because of their abundant expression of a copper-sequestering protein, metallothioneins (MTs) [30]. Copper ions are eventually released from astrocytes and then received by neurons [31] within the spinal cord and brain, which notably exhibit much slower turnover of copper ions than any other organs [32].

The intracellular level of copper ions is tightly regulated by a copper trafficking system composed of copper influx, delivery, and efflux [33]. Copper ions are incorporated into the cell by membrane proteins, copper transporter 1 (CTR1) [34,35] and probably also divalent metal transporter 1 (DMT1) [36]. Excess copper ions in cells of the CNS are excreted mainly by a copper efflux pump, ATP7A, at the plasma membrane and/or a cytosolic vesicular compartment [37]. ATP7A also exists at the membrane of intracellular organelles such as trans-Golgi network, where they supply copper ions to the organelles [37].

Once entered into the cell, the copper ion is delivered to specific intracellular copper-requiring enzymes/proteins by copper chaperone proteins [38]. While no free copper ions have been considered to exist in the cell [39], it remains obscure how copper ions are transferred from CTR1/DMT1 to copper chaperones. In mammals, three copper chaperones have been extensively studied so far: HAH1 [40], CCS [41], and COX17 [42] for delivering a copper ion specifically to a P-type ATPase in the *trans*-Golgi network, SOD1 in the cytoplasm, and cytochrome *c* oxidase in the mitochondria, respectively. Those copper chaperones have been considered to work independently of each other; namely, HAH1 and COX17 are not involved in the copper supply to SOD1 [38]. Instead, CCS can supply a conserved disulfide bond as well as a catalytic copper ion specifically to SOD1 [43], both of which are essential to the enzymatic activity of SOD1. While a CCS-independent pathway for SOD1 activation *in vivo* has also been proposed [44], significant reduction in the SOD1 enzymatic activity has been observed in mice with genetic deletion of *CCS* [41].

## 4. Perturbation in Copper Homeostasis in Mice Expressing ALS-Mutant SOD1

Intracellular concentrations of SOD1 are considered to range from 10 to 100 μM [45,46], and SOD1 is one of the proteins with the highest affinity for copper ions in cells [47]. It is thus expected that expression levels of SOD1 impact the homeostasis of intracellular copper ions. Indeed, the total amounts of copper ions in the mouse spinal cord, a region the most affected by ALS, are significantly elevated by expressing SOD1 with D90A and G93A [48,49,50,51,52], both of which retain affinity for copper ions [12]. Notably, overexpression of wild-type human SOD1 in mice also increased total amounts of copper ions in the spinal cord at relatively older ages (400 days) [51]. Wild-type SOD1 is supposed to be non-pathogenic, but its overexpression in mice has been shown to exhibit significant neurotoxicity in the spinal cord and cause a major loss of motor neurons at ages of around 600 days [53]. Given that overexpressed SOD1s (WT, D90A, and G93A) can function as an efficient sink for copper ions due to their high copper affinity, it is well expected that the total amounts of copper ions increased in the corresponding transgenic mice. Nonetheless, copper levels outside the SOD1 active site (non-SOD1 Cu levels) were also found to be elevated in the spinal cords of those transgenic mice [51]. Also, interestingly, the elevation of the non-SOD1 Cu levels is highly correlated with the disease progression of the mice expressing human SOD1 with G93A mutation (hSOD1^G93A^) and is observed in spinal cords but not in the brain, a region less affected by ALS [51]. These observations hence imply pathological roles of abnormal copper accumulation in *SOD1*-ALS cases.

The elevation in non-SOD1 Cu levels has also been observed in the transgenic mice expressing human SOD1 with G85R and G127X mutations, both of which exhibit almost no affinity for copper ions [51]. Pathological accumulation of copper ions in the spinal cords of ALS-model mice is hence not simply due to the increased concentration of copper-bound forms of overexpressed SOD1; rather, the copper trafficking system would be most likely disturbed by the expression of mutant SOD1 proteins and then cause abnormal accumulations of intracellular copper ions. Indeed, regardless of the copper-binding ability of mutant SOD1 expressed in transgenic mice, expression of a copper importer, CTR1, and a copper exporter, ATP7A, was shown to increase and decrease in the spinal cords, respectively [50,51]. Such changes in the expression levels of CTR1 and ATP7A resulted in the accumulation of copper ions inside the cell [51]. While further studies are required to clarify a mechanism of how mutant SOD1 changes the expression level of CTR1 and ATP7A, increased expression levels of copper-sequestering proteins, MTs, in the spinal cords of hSOD1^G93A^ mice also support the copper accumulation in the cells [54,55,56]. The expression level of MTs has been well-known to be transcriptionally up-regulated by the exposure to heavy metal ions [57], because multiple copies of metal-responsive elements are present in the promoter/enhancer regions of the *MT* genes [58]. Collectively, therefore, disturbance in the homeostatic control of intracellular copper ions is considered as a pathological hallmark in rodent models of *SOD1*-ALS.

## 5. Copper Homeostasis in Human ALS

Perturbation on the intracellular concentrations of copper ions would be involved in neurodegeneration; missense mutations in the copper efflux pump, ATP7A, have been identified to cause progressive X-linked spinal muscular atrophy type 3 [59], and the mice with the targeted knockout of ATP7A in motor neurons also exhibited motor neuron degeneration [60]. Indeed, a significantly increased level of copper ions as well as the other metal ions such as lead and zinc ions was reported in ventral areas of spinal cords from sporadic ALS cases [61], although mutations in *SOD1* gene have not been reported/confirmed in those cases.

In contrast, however, copper levels have not been reported in *SOD1*-ALS cases, and little consensus has been reached on any changes of the proteins maintaining homeostasis of intracellular copper ions in human ALS cases. For example, histopathological examination of fALS cases with SOD1 mutations (three cases with A4V and two cases with L126Z) has shown that CCS is co-aggregated with mutant SOD1 in the neuronal Lewy body-like hyaline inclusions in the spinal cords [62]. Another report has, however, described no staining of inclusions with anti-CCS antibody in a fALS case with A4V mutation as well as most of sporadic ALS cases [63]. Pathological changes in the expression level of MTs are also controversial: increased [64] or decreased [65] immunoreactivity to MTs was reported in several ALS cases. Collectively, it remains to be established whether any abnormalities in the homeostatic control of copper ions describe the etiology of human ALS cases. In particular, much more numbers of *SOD1*-ALS cases are definitely required to be examined for any abnormalities in the copper homeostasis.

## 6. Potential Therapeutics of *SOD1*-ALS by Lowering Intracellular Copper Levels by Small Compounds

Unlike human ALS cases, evidence of copper dyshomeostasis in mouse models of ALS is more convincing as described above; therefore, efficacy of a copper-lowering therapy to ameliorate the disease symptoms has been evaluated using animal models. Abnormal accumulation of copper ions in the liver and the brain is a main feature of an autosomal recessive disease, Wilson disease [66], and the drugs prescribed for Wilson disease include three copper chelators, d-penicillamine [67], trientine [68], and tetrathiomolybdate (TTM) [69], which can chelate and hence reduce the accumulated copper ions. So far, these three copper chelators, d-penicillamine [70], trientine [71,72], and TTM [51,73], have been also tested as potential drugs to normalize the intracellular copper levels and suppress the disease symptoms in hSOD1^G93A^ mice (Table 1). Treatment of hSOD1^G93A^ mice with TTM has been shown to restore abnormally increased concentrations of copper ions to a normal level in the spinal cord [51,73], but it has not been reported whether the other two chelators alter the intracellular levels of copper ions in the transgenic mice [70,71,72]. In any case, all of those three copper chelators were found to delay the disease onset and extend the lifespan of hSOD1^G93A^ mice, and importantly, TTM was effective in slowing the disease progression even when administered after the disease onset (Table 1) [51]. In other words, the therapeutic benefit of TTM was not affected by the timing to start the treatment [51,73].

It should also be noted that each of those chelators exhibited distinct effects on the disease duration; TTM markedly prolonged the disease duration in hSOD1^G93A^ mice, but d-penicillamine/trientine did not (Table 1). Such distinct efficacy among those three chelators would be described by their pharmacological properties [74]. That is, d-penicillamine and trientine are known to poorly cross the BBB and are also not permeable to the cell membrane [74]; therefore, those two chelators could remove copper ions from extracellular spaces but not directly modify the copper homeostasis inside the cell. Actually, a clinical study has shown little improvement with d-penicillamine therapy in five ALS patients when the drug was administered after appearance of several ALS symptoms [75]. While no information was provided as to whether those five ALS cases had mutations in the *SOD1* gene, therapeutic benefits of d-penicillamine might be obtained only before disease onset.

In sharp contrast, TTM is a membrane-permeable complex that can cross the BBB [76] and reach the CNS even by peripheral administration [73]; however, no clinical trial of TTM on ALS cases have not been reported. It is important to test if TTM is commonly effective in transgenic mice expressing mutant SOD1s other than G93A (e.g., G37R, G85R, L126Z). There is also a caveat that many other drugs showing 10%–20% improvement in the onset and/or the survival of transgenic mouse models have not been effective in the clinical trials [77]. TTM might be a candidate for drugs that could ameliorate the disease progression even after the disease onset, but we also need to modify TTM to increase efficacy to the ALS-model mice. Taken together, abnormalities in homeostatic control of intracellular copper ions could be corrected by administration of copper chelators, by which the disease progression of *SOD1*-ALS may be suppressed.

## 7. Proteins Regulating Intracellular Copper Levels as Potential Therapeutic Targets of *SOD1*-ALS

Not only do copper chelators modulate the intracellular concentration of copper ions, but proteins in the copper trafficking system can also control it. For example, copper ions are absorbed from small intestine *via* ATP7A; mutations in *ATP7A* gene, hence leading to the inhibited absorption of copper ions and the severe copper deficiency in Menkes disease [78]. Actually, a mouse possessing the X-linked mottled/brindled (Mobr) mutation, which is a model of Menkes disease [79], exhibits severe copper deficiency with lethal phenotypes similar to those of Menkes disease. Quite interestingly, when a fALS-model mouse expressing murine SOD1 with G86R mutation was crossed with a copper-deficient Mobr mouse, the survival was found to become prolonged with protection of motor neurons [80] (Table 2). These results are thus consistent with pathogenic roles of abnormally accumulated copper ions in fALS model mice expressing mutant SOD1 proteins.

Controlling the intracellular copper levels will also be possible by modulating expression of MTs because of their roles as an effective sink for free copper ions. MTs comprise a family of Cys-rich heavy metal-binding proteins with low molecular weight (<10 kDa). Human MT-I and MT-II, which are common MT isoforms, are composed of 61 amino acids, in which 20 Cys residues and no aromatic/hydrophobic residues exist [81]. Such a high content of Cys residues provides thiolate ligands for coordination of monovalent and divalent *d*^10^ metal ions including xenobiotic (cadmium, mercury, and silver) as well as physiological ones (copper and zinc) [81]. *In vitro* reconstitution experiments have shown that one MT molecule can tightly bind 12 atoms of copper with the stability constants ranging from 10^19^ to 10^17^ [81]. It is also important to note that neurons express not MT-I/-II but another MT isoform, MT-III [82,83]; MT-I/-II are actually expressed in glia. MT-III has the primary structure (68 a.a.) about 70% identical to that of MT-I/-II and can also bind heavy metal ions. Unlike MT-I/-II, MT-III was found to be secreted [84] and hence considered to play roles in extracellular metal-related neurochemistry. Given that MT-III can bind copper ions [85], the down-regulation of MT-III in a patient as well as a transgenic mouse model of Alzheimer’s disease has been proposed to alter copper homeostasis in the brain and then lead to extracellular amyloid pathology [86,87]. Important roles for MT-I/-II/-III proteins in copper metabolism of the brain and the spinal cord are hence well expected.

Indeed, ablation of either *MT-I/-II* or *MT-III* gene in hSOD1^G93A^ mice has been shown to exhibit significant reductions in survival [55,88] with further accumulation of copper ions in the spinal cord [89] (Table 2). Instead, a double transgenic mouse overexpressing MT-I and human SOD1 with G93A mutation exhibited a normal level of copper ions in spinal cords and showed prolonged survival with significant suppression of motor neuron death [54] (Table 2). Effects of MT-III expression on hSOD1^G93A^ mice were also explored by using a retrograde viral delivery system [90]. Even when injection of the adenovirus encoding *MT-III* gene started at the mean age of disease onset in hSOD1^G93A^ mice (~20 weeks), MT-III expression was found to prevent further loss of motor neurons and prolong the lifespan. These results thus suggest that metallothioneins play protective roles against the onset and progression of *SOD1*-ALS by controlling the copper metabolism.

Modulation of the MT-I/-II expression levels by administration of chemical drugs is also effective to ameliorate several disease phenotypes of ALS model mice. While multiple copies of the metal-responsive elements in the promotor of *MT-I/-II/-III* genes [58], zinc ion can induce the expression of MT-I/-II but not MT-III [83]. Indeed, oral intake of zinc ions induces MT-I/-II proteins in the small intestine, suppresses absorption of copper ions from the intestine [91], and thereby treat Wilson disease, a metabolic disease with abnormal accumulation of copper ions in the liver and brain. Against our expectations, however, a high dose of zinc supplementation has been reported to exacerbate the disease course in hSOD1^G93A^ mice [92]. While expression of MT-I/-II was not examined, a mild dose of zinc supplementation appears to have a tendency to prolong survival of hSOD1^G93A^ mice albeit with no statistical significance [93]. A dosage of zinc administration needs to be adjusted for its therapeutic benefits; however, zinc ions appear not to be effective to ameliorate the disease phenotypes.

Because of a glucocorticoid response element in the promotor region [94], a synthetic glucocorticoid, dexamethasone, is also known to induce expression of MT-I/-II [94] but not MT-III [83]. Consistently, treatment of hSOD1^G93A^ mice with dexamethasone was found to normalize the elevated levels of copper ions to a normal level in the spinal cord, and significantly prolonged survival as well as slowed disease progression even when the treatment was started at a symptomatic stage of the disease [89] (Table 2). Also, dexamethasone was not effective in hSOD1^G93A^ mice when the *MT-I/-II* genes were knocked out (Table 2), which confirms that therapeutic benefits by dexamethasone are mediated *via* expression of MT-I/-II [89]. Dexamethasone has been used to treat inflammatory conditions such as rheumatoid arthritis [95,96] and it might, therefore, be worth conducting a clinical trial on *SOD1*-ALS patients.

## 8. SOD1 Maturation as a Potential Therapeutic Target for *SOD1*-ALS

As mentioned, overexpression of SOD1 proteins leads to the abnormal accumulation of copper ions in the spinal cords of ALS-model mice [48,49,50,51,52], but, paradoxically, large fractions of mutant SOD1s in the spinal cords are considered to exist as a copper-deficient state. Although some mutant SOD1s (e.g., G37R, D90A, and G93A) have been shown to retain the enzymatic activity *in vitro*, which is comparable to that of wild-type SOD1 [10], most SOD1 proteins isolated from spinal cords of the transgenic mice were enzymatically inactive; based on the observed SOD1 activity, only about 25% of total SOD1 proteins appeared to be in a copper-bound form [12,48,51,97]. Upon the dissociation of metal ions, SOD1 has been shown to significantly reduce the thermal/structural stability and thus become susceptible to misfolding [98,99]. Although non-SOD1 Cu levels are abnormally elevated in transgenic mice overexpressing SOD1 proteins [48,49,50,51,52], such accumulated copper ions might exist as a form(s) that is somehow unavailable for SOD1. Accordingly, facilitating the supply of a copper ion to the active site of SOD1 could prevent the protein misfolding and thus be an alternative way to reduce the pathogenicity of SOD1. A daily supplementation of a copper-enriched diet, however, failed to increase the SOD1 activity and improve the disease phenotypes of hSOD1^G93A^ mice [12] (Table 3). This is probably due to tight regulation of bioavailable copper concentrations, and increased levels, if any, of intracellular copper ions do not simply facilitate the enzymatic activation of SOD1.

A copper chaperone, CCS, is well known to supply a copper ion specifically to SOD1 and thereby enzymatically activates SOD1 in the cell [43], while CCS-independent activation of SOD1 is also evident [100]. Simple overexpression of CCS in transgenic mice with mutant SOD1 would hence be expected to prevent the misfolding of copper-deficient SOD1 by facilitating the maturation of SOD1 proteins and, thereby, rescue the ALS-like symptoms; however, this was not the case. Rather, overexpression of CCS either aggravated the disease phenotypes of transgenic mice carrying G37R and G93A SOD1 or did not alter the disease course of mice expressing G86R and L126Z SOD1 (Table 3) [101,102]. Contrary to our initial expectation, Culotta and co-workers have shown that immature, misfolded forms of mutant SOD1 are actually increased in spinal cord of hSOD1^G93A^ mice by overexpression of CCS [106]. An apo-form of CCS was further found to interact with immature mutant SOD1 and probably inhibit the activation process of SOD1 [106]. Little pathological effects of CCS overexpression on G86R and L126Z mice would be due to severe structural destabilization of those mutant SOD1s [98,99], which hampers the interaction with CCS. Given that mutant SOD1 largely remains copper-deficient even with the abnormal elevation of non-SOD1 Cu levels [12,48,51,97], furthermore, overexpressed as well as endogenous CCS appears to be inaccessible to those accumulated copper ions and thus remains apo and probably trapped by immature mutant SOD1. Very recently, administration of the copper complex diacetylbis(*N*(4)-methylthiosemicarbazonato) copper(II) (Cu^II^(atsm)) has been shown to significantly improve severe phenotypes of the double transgenic mice expressing CCS and G93A SOD1 [97]. In addition to the removal of abnormally accumulated copper ions, supplying copper ions in a form(s) available for SOD1 and/or CCS would also be an effective therapy for *SOD1*-ALS.

Even in the absence of CCS overexpression, furthermore, oral treatment of mice expressing G37R or G93A SOD1 with Cu^II^(atsm) has been found to significantly delay onset of paralysis and extend the lifespan [103,104,105]. Also, the copper complex was found to be effective even when administered to symptomatic animals [103,104,105] (Table 3). Unlike the supplementation of copper-enriched diet, the hSOD1^G93A^ mice exhibited significantly increased activity of SOD1 by taking the Cu^II^(atsm) complex. This copper complex readily crosses the BBB with high membrane-permeability [107,108] and is supposed to facilitate the CCS-dependent activation of mutant SOD1 within the cell; in that sense, it will be interesting to test if the disease symptoms of ALS model mice can be ameliorated by the supplementation of Cu^II^(atsm) even in the absence of *CCS* gene. Also, in order to understand the pharmacological mechanism of Cu^II^(atsm) in *SOD1*-ALS model mice, its efficacy should be evaluated in the transgenic mice expressing G85R and L126Z SOD1, in which the copper-affinity of SOD1 is significantly compromised. Importantly, Cu^II^(atsm) is less toxic because it has been already used as a positron emission tomography-imaging agent for tumors in humans [109] and also for ALS patients [110]. More notably, administration of Cu^II^(atsm) to ALS-model mice was found to significantly reduce abnormal phosphorylation and truncation of TDP-43 [103], both of which are pathological hallmarks of sALS cases without mutations in SOD1.

Collectively, pharmacological and genetic modulation of intracellular copper ions have been found to affect the disease phenotypes of transgenic model mice of *SOD1*-ALS. In the ALS-model mice, copper ions are abnormally accumulated but unavailable for CCS and/or SOD1. In our opinion, therefore, the most preferable and efficient way to correct the intracellular copper homeostasis is to find drugs that can chelate the abnormally accumulated copper ions and then transfer those to CCS and/or directly to apo-SOD1. For that purpose, it will be important in the future to characterize how and where copper ions are accumulated in the spinal cord of model mice.

## 9. Concluding Remarks

We have reviewed recent advances in our understanding of copper dyshomeostasis in the pathogenesis of *SOD1*-ALS. More extensive and systematic investigation will be definitely required on possible involvement of copper dyshomeostasis in the *SOD1*-ALS cases. Regardless of the copper affinities of mutant SOD1 proteins, however, abnormal accumulation of copper ions in the spinal cord is considered as a pathological change in transgenic mouse models of *SOD1*-ALS. Indeed, removal of the abnormally accumulated copper ions by copper chelators or MTs was effective to ameliorate disease phenotypes. Even in the intracellular environment with abnormally accumulated copper ions, however, most fractions of mutant SOD1 in spinal cords are considered to exist as a copper-free form. In addition to the removal of abnormally accumulated copper ions, therefore, facilitation of copper loading into SOD1 proteins would also be taken into account for therapeutic development for *SOD1*-ALS. The most preferable method to reduce potential toxicities caused by copper dyshomeostasis is to facilitate the transfer of the abnormally accumulated copper ions into the metal-deficient forms of SOD1. Albeit paradoxically, the copper chelator, TTM, and the recently developed Cu^II^(atsm) complex all exhibited among the highest pharmacological benefits for transgenic mice expressing mutant SOD1 proteins; therefore, normalizing copper dyshomeostasis in pathological conditions will be a key to developing therapeutics for this devastating disease.

## Figures and Tables

**Table 1:** Treatment of hSOD1^G93A^ mice with copper chelators.

Treatment	Onset	Survival	Duration	Cu Level and SOD1 Activity
d-penicillamine (100 mg/day, p.o.) Pre-onset ([70])	↑ 8%	↑ 8%	No change	N.D.
Trientine (800 mg/day, p.o.) Pre-onset ([71])	Delayed ^1^	↑ 8%	No change	N.D.
Trientine (150 mg/day, p.o.) Post-onset ([72])	N.A.	No change	No change	N.D.
TTM (5 mg/day, i.p.) Pre-onset ([73])	↑ 20%	↑ 24%	↑ 42%	↓ Spinal Cu level ↓ SOD1 activity
TTM TTM (5 mg/day, i.p.) Post-onset ([51])	N.A.	↑ 11%	↑ 40%	↓ Spinal Cu level ↓ SOD1 activity

^1^ Based upon locomotion activity measured with a rota-rod apparatus. p.o., *per os* (orally); i.p., intraperitoneally; N.A., not applicable; N.D., not determined; ↓, decrease; ↑, increase.

**Table 2:** Modulation of intracellular copper levels in model mice of ALS.

Treatments	SOD1 Mice	Onset	Survival	Duration	Cu Level and SOD1 Activity
Crossed with Mobr mice ([80])	G86R	N.D.	↑ 9%	N.D.	↓ Spinal Cu level ↔ SOD1 activity
Crossed with *Mt-I/-II* knockout mice ([55])	G93A (low copy)	↓ 20%	↓ 13%	N.D.	N.D.
Crossed with *Mt-I/-II* knockout mice ([88])	G93A (low copy)	Accelerated ^1^	↓ 15%	N.D.	N.D.
Crossed with *Mt-I/-II* knockout mice ([89])	G93A (high copy)	↓ 17%	↓ 23%	↓ 34%	↑ Spinal Cu level ↔ SOD1 activity
Crossed with *Mt-III* knockout mice ([88])	G93A (low copy)	Accelerated ^1^	↓ 20%	N.D.	N.D.
Crossed with murine MT-I overexpressed mice ([54])	G93A (high copy)	↑ 8%	↑ 18%	↑ 57%	↓ Spinal Cu level ↔ SOD1 activity
Rat *Mt-III* DNA (1.0 × 10^9^ p.f.u.) Post-onset ([90])	G93A (high copy)	N.A.	↑ 11%	↑ 73%	N.D.
Zinc supplementation (375 mg/kg, p.o.) Pre-onset ([92])	G93A (high copy)	↓ 5%	↓ 8%	↓ 15%	N.D.
Zinc supplementation (18 mg/kg, p.o.) Pre-onset ([93])	G93A (high copy)	No change	No change	N.D.	N.D.
Dexamethasone (2 mg/kg, i.p.) Pre-onset ([89])	G93A (high copy)	↑ 8%	↑ 16%	↑ 37%	↓ Spinal Cu level ↔ SOD1 activity
Dexamethasone (2 mg/kg, i.p.) Post-onset ([89])	G93A (high copy)	N.A.	↑ 12%	↑ 89%	↓ Spinal Cu level ↔ SOD1 activity

^1^ Based upon grip strength and stride length. p.f.u., plaque forming unit; p.o., *per os* (orally); i.p., intraperitoneally; N.A., not applicable; N.D., not determined; ↔, no change; ↓, decrease; ↑, increase.

**Table 3:** Modulation of the copper-binding status of SOD1 in model mice of ALS.

Treatments	SOD1 Mice	Onset	Survival	Duration	Cu Level and SOD1 Activity
Cu diet (400 ppm/day, p.o.) Pre-onset ([12])	G93A (high copy)	N.D.	No change	N.D.	↔ Spinal Cu level ↔ SOD1 activity
*Ccs* knockout ([100])	G93A (high copy) G37R G85R	No change	No change	No change	↔ Brain Cu level ↓ SOD1 activity
Human CCS overexpression ([101])	G93A (high copy)	Accelerated ^1^	↓ 85%	N.D.	N.D.
Human CCS overexpression ([102])	G37R	Accelerated ^1^	↓ 90%	N.D.	N.D.
Human CCS overexpression ([102])	G86R L126Z	No change	No change	No change	N.D.
Cu^II^(atsm) (30 mg/day, p.o.) Pre-onset ([103])	G93A (low copy)	↑ 8%	↑ 14%	↑ 70%	↑ SOD1 activity
Cu^II^(atsm) (30 mg/day, p.o.) Post-onset ([103])	G93A (low copy)	N.A.	↑ 10%	↑ 59%	↑ SOD1 activity
Cu^II^(atsm) (60 mg/day, p.o) Pre-onset ([104])	G37R	Delayed ^2^	↑ 26%	N.D.	N.D.
Cu^II^(atsm) (60 mg/day, p.o) Post-onset ([104])	G37R	N.A.	↑ 12%	↑ 43%	N.D.
Cu^II^(atsm) (30 mg/day, p.o.) Pre-onset ([105])	G37R	Delayed ^1^	N.D.	N.D	↑ Spinal Cu level ↑ SOD1 activity

^1^ Based upon grip strength and stride length; ^2^ Based upon locomotion activity measured with a rota-rod apparatus. p.o., *per os* (orally); N.A., not applicable; N.D., not determined; ↔, no change; ↓, decrease; ↑, increase.

## Abbreviations

ALS	amyotrophic lateral sclerosis
BBB	blood-brain barrier
CCS	copper chaperone for SOD1
CNS	central nervous system
CTR1	copper transporter 1
DMT1	divalent metal transporter 1
fALS	familial ALS
MTs	metallothioneins
sALS	sporadic ALS
SOD1	Cu,Zn-superoxide dismutase
*SOD1*-ALS	ALS with mutations in *SOD1* gene
TTM	tetrathiomolybdate
